# MicroRNA miR-425 promotes tumor progression by inhibiting Dickkopf-related protein-3 in gastric cancer

**DOI:** 10.1080/21655979.2021.1930743

**Published:** 2021-06-14

**Authors:** Yihua Pei, Zhiteng Tang, Minjing Cai, Qin Yao, Bozhen Xie

**Affiliations:** a Central Laboratory, ZhongShan Hospital XiaMen University, Xiamen, Fujian, China; b Department of Pathology, ZhongShan Hospital XiaMen University, Xiamen, Fujian, China; c Department of Center of Clinical Laboratory, ZhongShan Hospital XiaMen University, Xiamen, Fujian, China; d Department of Spine Surgery, ZhongShan Hospital XiaMen University, Xiamen, Fujian, China

**Keywords:** MiR-425, DKK-3, gastric cancer, oncogene

## Abstract

Gastric cancer is a prevalent yet heterogeneous disease which ranks as the fifth most common cancer in the world. Dietary habit, genetic background, *Helicobacter Pylori* infections were the risk factors of gastric cancer. MicroRNA miR-425 is highly expressed in gastric cancer, but little attention has been devoted to the mechanism of miR-425 in tumorigenesis. This study aim to investigate the role of miR-425 in gastric cancer.

The expression of miR-425 and Dickkopf-related protein-3(DKK-3) were analyzed by qRT-PCR. Gastric cell line BGC-823 and SGC-7901 were transfected miR-425 inhibitors or NC. Then, cell viability was determined by CCK-8, cell apoptosis and cell cycle were assessed by flow cytometer. Cell migration and cell invasion were analyzed by wound healing and trans-well assays. Luciferase reporter assay was conducted to assess the correlation between miR-425 and DKK-3. Downstream regulators, such as p-ASK1 and p-JNK, were analysis by western blot.

Compared with normal gastric epithelium cell line, miR-425 was obviously upregulated in gastric cancer cell lines. MiR-425 inhibitor suppressed the cell viability, cell migration and cell invasion. The Luciferase assay data identified that DKK-3 is a target of miR-425. While miR-425 could lower the expression of DKK-3 which mediate tumorigenesis in a certain way.

## Graphical abstact

**Figure:**
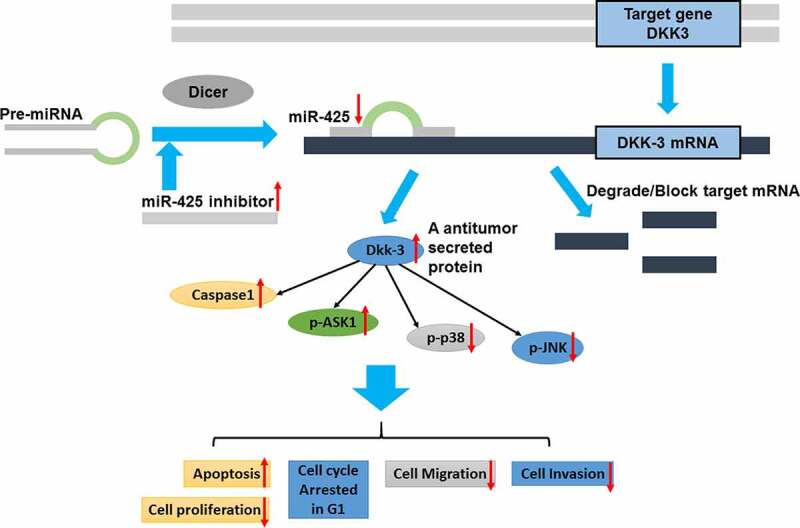

## Introduction

There are over 1 million new gastric cancer cases estimated each year world widely [1,2]. Gastric cancer is a prevalent yet heterogeneous disease, with *Helicobacter pylori* infection, dietary habit, genetic background were the main risk factors. In clinical, gastric cancer is diagnosed histologically after laparoscopy, staged CT, endoscopic biopsy, endoscopic ultrasound and PET. For early-stage gastric cancer, endoscopic resection (ER) is the most important and valid treatment [3]. For advanced gastric cancer, comprehensive treatment regimens including chemotherapy, surgery, immunotherapy and target therapy [4], but the median survival time was still less than 1 year after treatment. Though there are continuous improvements in the technologies of diagnosis and treatment, current treatments for advanced gastric cancer are very limited because of the distant metastasis. Thus, revealing the mechanisms of tumorigenesis in gastric cancer is urgently needed.

It has been demonstrated that DKK-3 belongs to the Dickkopf family that implicated in cell fate decision and embryonic development. The DKK-3 located in 11p15.3(NC_0000111.10, 11,963,036 … 12,009,827). Dkk-3 mediates potent antitumor effects through binding extracellular receptor Wnt/β-catenin. Besides, DKK-3 is highly expressed in heart, brain and spinal cord, it could reduce cell proliferation, metastasis and anchorage-independent growth. Accumulating evidence indicates that gastric cancer has lower levels of DKK-3 and its methylation is a very common event in gastric and colorectal cancers [5]. Previous studies have indicated that DKK-3 act as a crucial role in carcinogenesis.

MicroRNAs(miRNAs) are small non-coding RNAs with length of 18–25 nucleotides. MicroRNAs function as a regulator of posttranscriptional, it could inhibit messenger RNA(mRNA) from forming or facilitate mRNA degradation. In consideration of the diversity of target gene, miRNA may implicate in the regulation of diverse biological processes. Actually, previous studies have suggested that miRNAs mediate several pathologies including cell apoptosis, cell invasion and differentiation [6]. Abnormal expression of miRNAs may contribute to tumorigenesis with fine-tuning the expression of target gene. For instance, high miR-129 expression could repress CP110 which may regulate the metastasis of prostate cancer [7]. While forced miR-26a expression downregulates the expression of PTEN which lead to promote hepatocellular carcinoma migration and invasion [8]. In addition, the shorter progression-free survival of colorectal cancer may relate to the cell growth inhibitor effect of miR-193 [9]. These findings have highlighted that miRNAs potentially act as a new target for therapeutic strategies against gastric cancer. Nowadays, only few publications have noticed that inhibiting miR-425 could inhibit the cell migration and invasion via DKK-3. Hence, the pathologic mechanism of miR-425 and its correlation with DKK-3 in gastric cancer is urgent to figure out.

According to the available literatures, in this article, we hypothesis that miR-425 may be an oncogenic miRNA in gastric cancer. We aimed to provide some insight into the role of miR-425 in the tumorigenesis of gastric cancer, and try to investigate the mechanism which may allow for the discovery of new therapy target.

## Materials and methods

### Cell culture

MGC-803, GES-1, BGC-823 and SGC-7901 were purchased from ATCC. Cells were cultured in RPMI-1640 (Gibco, USA) complete medium which contains 10% FBS, penicillin-streptomycin. The cultivation condition is 37°C, 5% CO_2_. The mycoplasma pollution should be tested every 3 months. All the cells were collected during the logarithmic growth phase for subsequent experiments.

### Quantitative real-time Polymerase chain reaction

Total RNA was isolated by Takara MiniBEST universal RNA Extraction Kit (Takara, Japan). The cDNAs were reverse transcribed using a PrimeScript RT Reagent Kit witg gDNA Eraser (Takara, Japan). miR-425 sequences: AAUGACACGAUCACUCCCGUUGA(MIMAT0004750, miRBase). qPCR was performed using the SYBR-Green SuperMix kit (Bio-Rad, USA) in real-time PCR System (Bio-Rad). Protocol followed: 95°C for 10 min, 38 cycles of 94°C for 12 seconds, 58°C for 20 seconds, and 72°C for 14 seconds. Set GAPDH and U6 were the control group. Primer sequences are listed as follows:

**Table:** 

Gene	Primer sequences (5ʹ-3ʹ)
DKK-3	Forward primer: CTGTGTGTCAGGGGTCACTG
	Reversed primer: GCTCTAGCTCCCAGGTGATG
GAPDH	Forward primer: ACAGCAACAGGGTGGTGGAC
	Reversed primer: TTTGAGGGTGGCAGCGAACTT
miR-425	Forward primer: AAUGACACGAUCACUCCCGUUGA
	Reversed primer: CCAGUGCUCGACUCAUCGCGGCG
U6 snRNA	Forward primer: CTCGCTTCGGCAGCACATATACT
	Reversed primer: ACGCTTCACGAATTTGCGTGTC

### Plasmid construction and transfection

Cells were cultured in 12-well plates with complete medium. After Grow the cells to 70% confluence, BGC-823 and SGC-7901 cell lines were transfected with miR-425 inhibitor and negative control (Biossci, China) using Lipofectamine 2000 (Sigma-Aldrich, CA, USA). After 4 h, add 500 μl complete medium to each well.

### Cell viability assay

Following transfection, MGC-803, SGC-7901 were planted in 96-well plate with 4000 cells/well. All cells were incubated at 37°C, 5% CO_2_. Using Cell Counting Kit-8 (MCE, USA) to assess cell viability. The absorbance spectrum was tested at 450 nm with a microplate reader (Mithras LB943, USA).

### Cell cycle analysis and apoptosis

For cell cycle, cells were harvested and resuspended in cold PBS after transfection. Then, immobilized cells in 70% ethanol and stored at 4°C overnight. After immobilization, the cells were stained in 1 mL of cold PI solution (50 μg/mL) containing 100 μg/mL RNase A. Incubation for 30 mins on ice in the darkness. Cell apoptosis ratios of were measured using FITC Annexin V Apoptosis Detection Kit (BD Pharmingen, USA). Both cell cycle and cell apoptosis were performed by LSRFortessa flow cytometer (BD, USA). At least 20,000 cells were recorded for the analysis and all data were exported and re-analyzed by FlowJo 10.0.7 software (Tree Star, USA)

### Cell invasion and migration assays

Before performing cell invasion and migration assays, cells were starved for 12 h in serum-free RPMI media. Using 100 μL tips to generate the wounds in the confluent cell with density of 80%. At time 0 h and 24 h, the wound gap distances were photted under microscope (Olympus IX71, Japan). On the other hand, inserts were pre-coated with 1%BME and 24-well Corning BioCoat Matrigel Invasion Chambers (SLS Ltd., UK) were carried out to test cell invasion. Every insert was recorded at least five pictures at ×100 magnification using an IX71 microscope (Olympus). All pictures were analyzed by Image-Pro Plus (QImaging, Canada).

### Luciferase reporter assays

Luciferase reporter assays were performed to study whether DKK-3 gene expression involved in miR-425 regulation or not. DKK-3 wildtype and mutated-type reporter plasmids were constructed (Figure 3). In a 12-well plate, cells were transfected with triplicate for each group. After 6 h, add complete medium to each well to sustain culture. Using Luciferase Reporter Assay Kit (Abcam, USA) to measure luciferase activities.

### Western blot analysis

After 24 h since cell transfection, cell culture media were discarded and complete medium was added to each well. Before protein extraction, cell should be cultured in serum-free medium. Total cell proteins were collected and lysed in 300 µl RIPA (150 mM NaCl, 0.5% Na deoxycholate, 20 mM Tris pH 8.0, 0.1% SDS, and 1% Triton X-100) in a cold Eppendorf tube. Tubes were centrifuged under a condition of 4°C, 15,000 g, 15 mins. Bicinchoniic kit (Beyotime, China) was used to qualified protein concentration. 50ug of total protein was loaded in 12% SDS-PAGE and separated in 10% SDS-PAGE gels. Subsequently, membranes were transferred to electrophoretic ally transferred onto polyvinylidene fluoride. Block buffer is 5% skimmed milk, PVDF membranes were blocked for 2 h at 25°C. After blocking, wash the membranes twice and add primary antibodies lightly, all the primary antibodies used were as follows: DKK-3 (dilution 1:100; cat. no. 10,365-1-AP; Proteintech Group, USA), p-p38 (dilution 1:100; cat. no. 4511; Cell signaling, USA), p-ASK1 (dilution 1:100; cat. no. 3764; Cell signaling, USA), anti-caspase-1(dilution 1:100; cat. no. 3866; Cell signaling, USA), p-JNK (dilution 1:100; cat. no. 9251; Cell signaling, USA), incubated overnight at 4°C. Next day, discard the primary antibodies and wash twice in PBST, then add goat anti-rabbit IgG as secondary antibody to incubate for 2 h at 25°C. All membranes were visualized using Supersignal West Atto kit (Thermofisher, USA), and analyzed with ChemiDocXRS+ system (Bio-Rad Laboratories, US).

## Statistical analysis

Data were presented as means ± SD. Using two-tailed Student’s t test and χ2 test to assess P value. All experiments were repeated at 3 times, and groups were compared by One-way analysis of variance. Considering P < 0.05 is statistically significant. GraphPad Prism 6 (GraphPad Software, US) and SPSS 20.0 (SPSS, US) were used to analyse the statistical data.

## Result

First, we examined the expression of miR-425 in cell lines. Based on the expression of miR-425, cells were transfected with miR-425 inhibitor or control. Second, the proliferation, apoptosis and cell cycle were assessed by FACS. Third, cell immigration and invasion were performed to investigate the role of miR-425 in tumorigenesis. Then, Caspase-1, ASK-1, p-P38 and p-JNK were tested by western blot.

### MiR-425 was up-regulated in gastric cancer cell lines

**Figure 1.**
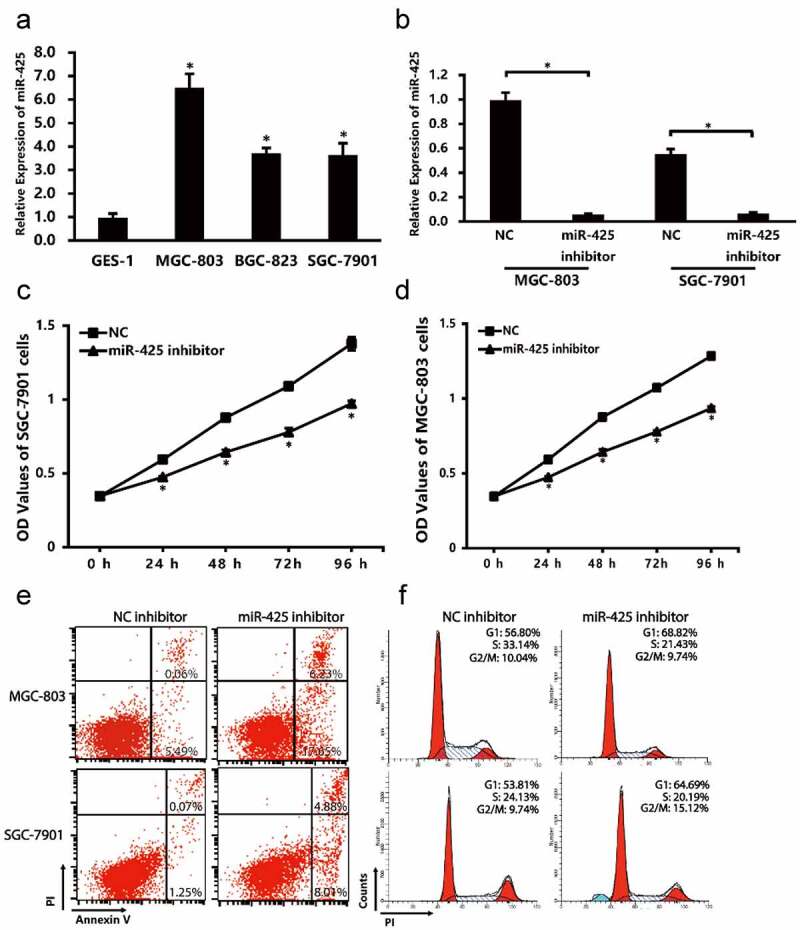
The expression of miR-425 in cell lines and the cell status after transfection. (a) miR-425 expression in different cell lines. (b) Transfection efficiency of miR-425 was verified by qRT-PCR. (c,d) cell proferation was assessed by CCK-8 every 24 h. (e, f) Cell apoptosis and cell cycle were performed by flow cytometry. All the date represent mean ± SD, and all experiments were repeated for 3 times. *P < 0.01 vs. control group

The expression levels of miR-425 in cell lines were analyzed by qPCR. As shown in Figure 1a, compare to normal gastric endothelial GES-1, miR-425 is highly expressed in MGC-803, BGC-823 and SGC-7901 cells.

### Validation of cell transfection efficiency

To examine the transfection efficiency, qRT-PCR was performed in MGC-803 and SGC-7901 cell lines. Compared with NC group, the expression of miR-425 is significantly lower in MGC-803 and SGC-7901 cells (Figure 1b).

### Downregulation of miR-425 affect cell proliferation, apoptosis and cell cycle

To explore the biological role of miR-425 in cell proliferation, apoptosis and cell cycle, CCK-8 assay and flow cytometry analyses were performed. After transfection with the miR-425 inhibitor, the proliferation of MGC-803 cells was decreased by 19.98% (24 h; P < 0.05), 26.73% (48 h; P < 0.05), 27.33% (72 h; P < 0.05), 27.16% (96 h; P < 0.05) compare with the negative control (Figure 1c). This decrease also takes place in SGC-7901 cells. The flow cytometry data showed that miR-425 inhibitor group has a higher apoptosis rate (23.88%) than negative control (8.17%) in MGC-803, the same result can be obtained in SGC-7901 cells (12.89% vs. 1.32%), which means downregulation of miR-425 may trigger apoptosis of gastric cancer cell. Following, the cell cycle data indicated that the miR-425 inhibitor group have a higher percentage of G1 phase (68.82% vs.56.80%; 64.69% vs. 53.81) compared to NC group. These clues suggest that downregulation of miR-425 may inhibit gastric cancer cell viability, promote cell apoptosis and arrest cell cycle on G1 phase.

### miR-425 promotes cell migration and invasion of MGC-803 and SGC-7901 cells

**Figure 2.**
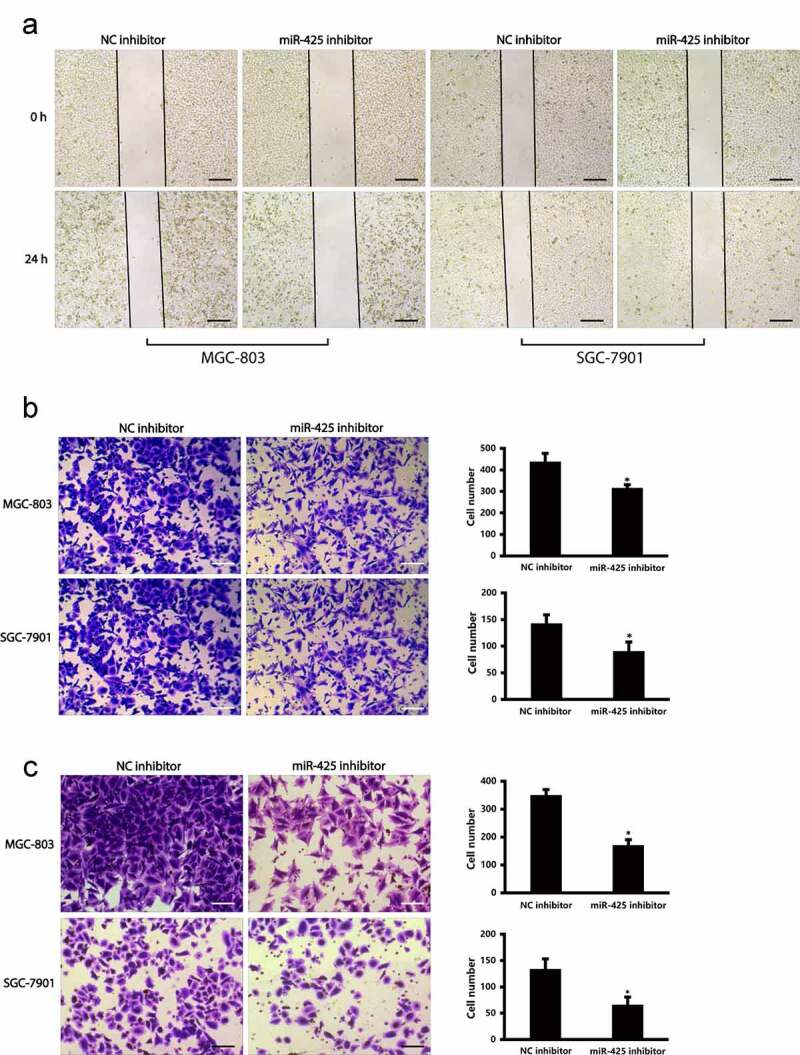
miR-425 promotes cell migration and invasion in MGC-803 and SGC-7901 cell lines. (a) Cell migratory ability was evaluated in MGC-803 and SGC-7901 cells in cell wound healing assay. Scale bars 200 μm (b) The trans-well migration assay result of migratory ability of MGC-803 cells and SGC-7901 cells after transfection. Scale bars 100μ. (c) The invasive ability of MGC-803 and SGC-7901 cells. (*p < 0.05 vs. NC inhibitor). Scale bars 100 μm

To reveal the biological role of miR-425 in cell migration and invasion, wounding assay and trans-well migration assay were conducted in MGC-803 and SGC-7901 cell lines. The photos of wound healing assay indicated that downregulation of miR-425 obviously holdback cell migration in MGC-803 and SGC-7901 cells (Figure 2a). While in trans-well assay, down-regulation of miR-425 holdback cell migration in MGC-803 and SGC-7901 cells (Figure 2b). Moreover, compared with control group, the invaded cells were lower in miR-425 inhibitor group (Figure 2c). All the photographs indicate that miR-425 could facilitate cell migration and invasion in gastric cancer cells.

### miR-425 negatively regulates DKK-3 expression by target binging

**Figure 3.**
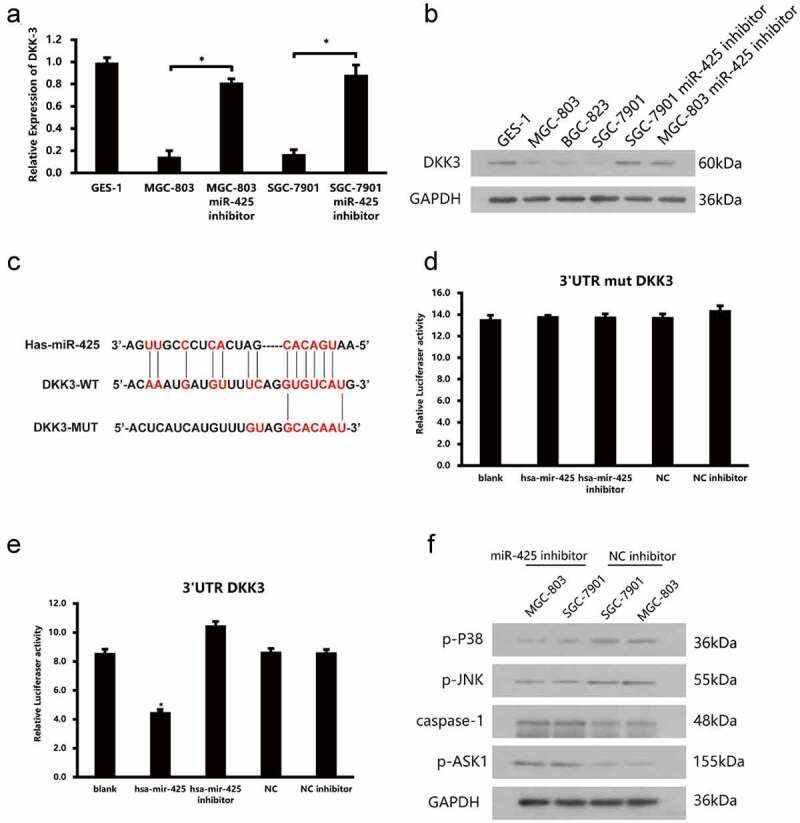
miR-425 negatively regulates DKK-3 expression by targeted combination. (a, b) The expression of miR-425 in GES-1, MGC-803 and SGC-7901 and cell lines after transfection. (c) Predicted binding sites between miR-425 and the 3ʹUTR sequence of DKK-3. (d, e) Relative luciferase activities of 3ʹUTR DKK-3 and 3ʹUTR mutant DKK-3. (f) Western blot analyses of p-P38, p-JNK, caspase-1, p-ASK1 in cells lines after transfection with miR-425 inhibitor (*p < 0.05 vs. NC inhibitor)

According to the results above, to investigate how the miR-425 come into function and what is its target, we first screened out a secretory protein DKK-3, which have been approved as an important regulator of tumorigenesis. Quantitative real-time PCR and western blot of miR-425 and DKK-3 were examined to check the expression. The data demonstrate that the expression of DKK-3 was obviously lower in gastric cancer cell lines than normal cells (Figure 3a, 3b). Combining the expression data of DKK-3 and miR-425, these data tell us that miR-425 maybe an upstream regulator of DKK-3. At first, we use online prediction website-Targetscan to scan the binding site of miR-425 to DKK-3. Then, we constructed a plasmid with the sequence of the 3ʹUTR of DKK-3 which contains the putative binding sites of miR-425 for luciferase reporter assay. On the other hand, mutant reporter vector was constructed as a negative control. The luciferase activity results revealed that downregulation of miR-425 increased the luciferase activity of DKK-3 3ʹUTR significantly. However, no luciferase activity on the mutated reporter vector (Figure 3d, 3e). Further, we tested the expression level of p-P38, p-JNK, p-ASK1, caspase-1 which were the downstream signaling molecular of DKK-3 (Figure 3f). It is interesting to note that upregulation of DKK-3 which caused by inhibiting miR-425 shows a significant elevated expression level of pASK1 and caspase-1. Meanwhile, p-P38 and p-JNK were decreased compared to negative control. In summary, these data proved that miR-425 could regulate the expression of DKK-3 negatively, which may influence the downstream signal molecular of DKK-3 in a certain way.

## Discussion

Over 1 million new gastric cancer cases were estimated each year world widely [1,2]. Gastric cancer is a prevalent yet heterogeneous disease, with *Helicobacter pylori* infection, dietary habit and genetic background are the main risk factors [10,11]. During the past 30 years, much more information has become available on gastric cancer. The serum pepsinogens, ghrelin and antibody to H. pylori were the diagnosis markers of earlier gastric cancer. The barium meal examination and endoscopy are very valid for diagnosis in clinical for now [12]. Actually, in high-risk population, screening mass populations for gastric cancer has been provided although the value of it remains controversial. That is to say, understanding the mechanism of gastric and discovering new therapy target is vital to the prevention of gastric cancer.

Given that more and more evidence has shown that a number of miRNAs are involved in tumor growth and migration, it acted as oncogenes or tumor suppressors during tumor progression. We hypothesis that miRNA may provide an interesting point of view to understand gastric cancer. Indeed, accumulation publications showed that the expression of miR-425 was altered in esophageal cancer, lung cancer and glial cancer [13–15]. miR-425 was up-regulated in hepatocellular carcinomas cells, and forced expression of miR-425 promoted proliferation and migration by inhibiting CTNNA3 hepatocellular carcinomas cell [16]. Besides, over-expression of miR-425 also promotes tumorigenesis by targeting SMAD2 in esophageal squamous cells [17]. However, the expression of miR-425 was lower in human melanoma compared to normal cells, it repressed the PI3K-Akt pathway by targeting IGF-1 lead to inhibits melanoma metastasis [18]. Those results suggested that the biological function of miR-425 may depend on the types of tumors. Recently, there is pivotal evidence that miR-425 was highly expressed in gastric cancer than adjacent mucosa [19].

To investigate the role of miR-425 in gastric cancer, in this study, we firstly confirmed the expression of miR-425 was highly expressed in gastric cancer cell lines than in normal gastric cell lines. Then, we inhibit miR-425 by transfecting specific inhibitor in MGC-803 cells and SGC-7901 cells. However, the proliferation, invasion, and migration of gastric cells were obviously decreased after inhibiting miR-425, while the apoptotic rates were elevated after transfection. On the contrary, downregulation of miR-425 arrest more cells in G0/G1 phase and less cells in G2/M and S phase. All these results indicated that miR-425 may function as a tumor promoter in gastric cancer.

To explore how the miR-425 promotes gastric cancer tumorigeneses and what is the target gene of it. Algorithms Targets can, and miRanda website tools were used to screen out the potential target gene of miR-425. Fortunately, DKK3 was one of the targets with high score. Given that Dkk3 is a tumor suppressor and its expression is significantly lower in a variety of human cancer types. Such as, mRNA and protein level of DKK3 were downregulated in colorectal adenocarcinoma cell lines. And the migration and invasion capacity were elevated after silencing DKK-3 expression by siRNA in colon cancer cells. What is more, downregulated Dkk3 result in a reduction in tube formation in Matrigel®, this reduction was reversed after enforced overexpression [20]. In melanoma mouse model, highly expressed DKK-3 could enhance the micro vessel density remarkable [21]. Based on the previous studies, we performed luciferase reporter assays and the data showed that miR-425 may target DKK3-3ʹUTR with the seed sequence directly. Western blot verified that suppress the miR-425ʹexpression could upregulate the expression of DKK3. All the above data suggested that DKK3 act as a potential functional target of miR-425. According to the previous research findings, DKK-3 may influence G1/G0 phase cell cycle arrest, cell apoptosis, and high cytoplasmic beta-catenin. To figure out which pathway the miR-425 and DKK3 may participate in, we detect the expression of p-P38, p-JNK, caspase-1 and p-ASK1 by western blot. The results show that p-P38, p-JNK seems decreased after transfecting miR-inhibitor. On the contrary, caspase-1 (a cysteine protease associated with cell apoptosis) and p-ASK1 which play an essential role in stress-induced apoptosis [22,23] were upregulated, which may explain the apoptosis rates triggered by miR-425-DKK3 axis. However, one of the major limitations of this study is that we paid special attention to the involvement of miR-425 to DKK-3 and that other potential mechanisms have not been investigated.

## Conclusion

In conclusion, our data indicated that miR-425 may function as an oncogene in gastric cancer cell lines. And miR-425 could regulate the cell viability, cell invasion and migration in a certain way.

Besides, caspase-1 and ASK-1 signal pathway, and MAPK pathways were involved in these processes of gastric cell. Taken together, miR-425 may function as an oncogene via restraining the expression of DKK-3.

## Supplementary Material

Supplemental Material

## Highlights:

MiR-425 was obviously upregulated in gastric cell lines.+

Downregulation of miR-425 could inhibit the cell proliferation and migration.

DKK-3 is a direct target of miR-425, involved in mediating of JNK/MAPK signaling pathway.
