# Supplementary figures and images for: MicroRNA miR-425 promotes tumor progression by inhibiting Dickkopf-related protein-3 in gastric cancer
Source: Bioengineered. 2021 Jun 14;12(1):2045–54. doi: 10.1080/21655979.2021.1930743 (PMC8806936; doi:10.1080/21655979.2021.1930743)

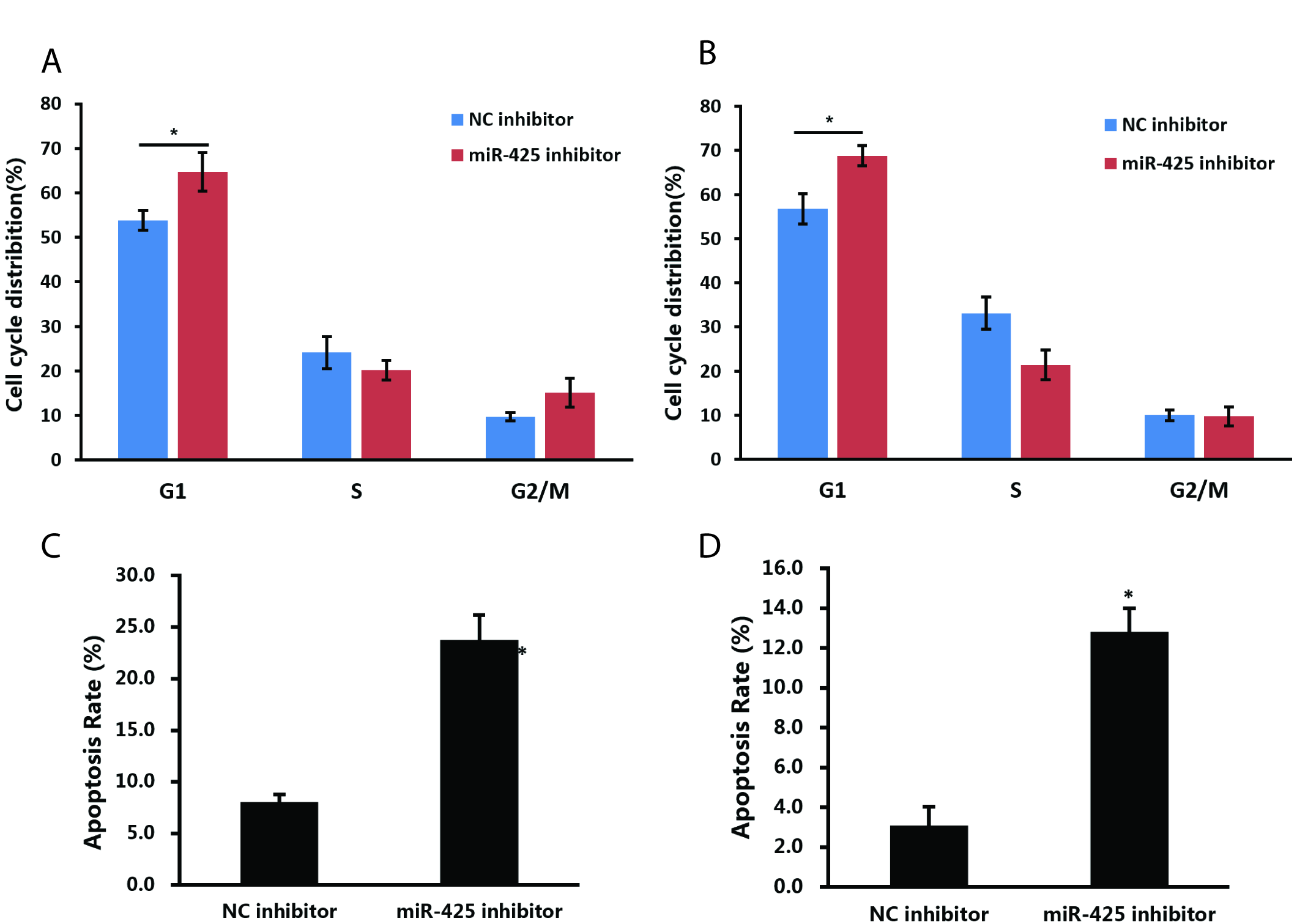

Supplement: Supplemental Material [file KBIE_A_1930743_SM8440.zip › supplementary/supplement 1.tif]

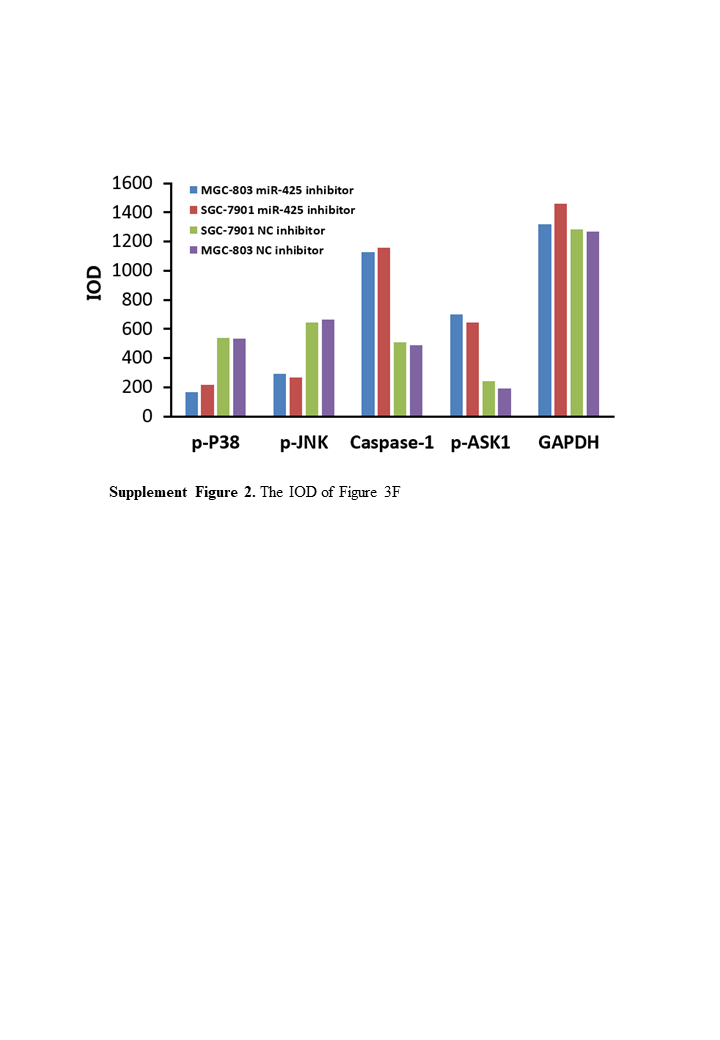

Supplement: Supplemental Material [file KBIE_A_1930743_SM8440.zip › supplementary/supplement figure 2.tif]
